# Boosted Chemical Protective Properties Using Interface Constructed between Ti_3_C_2_T_x_ MXene and Natural Rubber

**DOI:** 10.3390/polym15214260

**Published:** 2023-10-30

**Authors:** Qinyu Chen, Min Zhang, Xiaopeng Li, Chuan Zhou, Guang Yang, Heguo Li, Xiaohui Zheng

**Affiliations:** State Key Laboratory of NBC Protection for Civilian, Beijing 100191, China; cqy2998@163.com (Q.C.); minjohng@126.com (M.Z.); lxpbuct@163.com (X.L.); zhouc_fh@163.com (C.Z.); ygnudt@163.com (G.Y.)

**Keywords:** Ti_3_C_2_T_x_ MXene, nanocomposite, three-dimensional network, impermeability, mechanical property

## Abstract

Rubbers are extensively applied in chemical protective clothing (CPC) due to their eye-catching anti-penetration of chemicals. However, their impermeability, particularly that of natural rubber (NR), is unsatisfactory. In this work, we demonstrate the facile construction of Ti_3_C_2_T_x_ MXene/NR interface using a plant-scale and feasible method combining latex mixing, emulsion flocculation, and flat-plate vulcanisation. The above crafts achieved a homogeneous dispersion of Ti_3_C_2_T_x_ MXene in the NR matrix in a single layer, thereby constructing a strong interfacial interaction between Ti_3_C_2_T_x_ MXene and NR, which induced the formation of a robust three-dimensional (3D) network in the composite. The anti-swelling capacity of the 3D cross-linked network structure and the layered structure of Ti_3_C_2_T_x_ MXene effectively prolonged the permeation path of toxic chemicals. Compared with pure NR, the nanocomposite with 1 wt% of Ti_3_C_2_T_x_ MXene showed substantially enhanced breakthrough times of toluene, dichloromethane, and concentrated sulfuric acid (increased by 140%, 178.6%, and 92.5%, respectively). Furthermore, its tensile strength, elongation at break, and shore hardness increased by 7.847 MPa, 194%, and 12 HA, respectively. Taken together with the satisfactory anti-permeability, tensile strength, elongation at break, and shore hardness, the resulting Ti_3_C_2_T_x_ MXene/NR nanocomposites hold promise for application to long-term and high-strength CPC in the chemical industry and military fields.

## 1. Introduction

Frequent chemical accidents [1,2] or chemical attacks [3,4,5] heavily emphasize the significance of chemical protective clothing (CPC) [6,7,8].Chemical protective equipment is divided into four categories based on the various levels of protection as defined by the United States Environmental Protection Agency (USEPA), ranging from minimal dermal and respiratory protection (level D) to maximal protection (level A) [9]. Due to their anti-penetration, flexibility, and processability [10,11,12], rubbers are generally applied to encapsulating outfits for high-level chemical protection or chemical-resistant gloves and boots against various levels of toxic chemicals. Compared to synthetic rubber (SR), renewable and environmentally friendly natural rubber (NR) can be separated from common rubber trees, such as Hevea brasiliensis [13]. The protection ability of CPC is mainly determined via permeation, penetration, and degradation parameters [11,14]. However, NR only works for the penetration of chemicals in the form of liquid, vapor, and aerosol [11], its behaviours in anti-permeability [15] and anti-degradation of acids [16] are unsatisfactory. Therefore, it is essential to improve the protective performance of NR against toxic chemicals. Furthermore, its mechanical property is another vital aspect to guarantee the practical applications of NR for CPC [11], mainly including tear strength, tensile strength, cutting resistance, puncture resistance, and abrasion resistance.

Filling NR with the proper types of nanomaterials, such as carbon-based, inorganic-based, and bio-based fillers [17], can optimize its barrier property, mechanical performance, and other desirable properties. For instance, George et al. [18] claimed that multiwalled carbon nanotubes (MWCNT) nanofillers have a positive impact on the solvent resistance and gas barrier properties, as well as on the mechanical properties of NR-MWCNTR nanocomposites. The considerable mechanical behaviours of NR-based composites armed with carbon black or Al_2_O_3_ nanoparticles were reported by Nouraei et al. [19]. Wongwat’s [20] group prepared the NR/poly(lactic acid)/thermoplastic starch/nano-precipitated calcium carbonate (NR/PLA/TPS/NPCC) nanocomposites and reported that the NPCC content and mixing order of constituents affected the multiple performances of NR/PLA/TPS/NPCC nanocomposites, such as tensile and water vapor barrier properties. Although these fillers clearly improved the barrier or mechanical characteristics of NR, they were equipped with few surface functional groups and interacted with NR chains weakly, causing their aggregation within the NR matrix [21], thus failing to fully improve the mechanical or barrier performances of NR.

Because of their rich surface chemistry and high mechanical properties [22,23,24,25], two-dimensional (2D) transition metal carbide/nitride/carbonitride (MXene) sheets have been intensively and extensively reported as nanofillers to improve the mechanical performances of SR and chemical resistances of P84 copolyimide [26,27]. However, there has been no investigation related to MXene/NR in the chemical protective field to date. Considering that the NR obtained from rubber trees exists in the form of latex, which is an aqueous colloidal dispersion of rubber particles, Ti_3_C_2_T_x_ MXene nanofillers with strong hydrophilicity can be easily distributed in the NR matrix [21]. In this research, a Ti_3_C_2_T_x_ MXene/NR nanocomposite (MNx) was prepared via a feasible method combining latex mixing, emulsion flocculation, and flat-plate vulcanisation technologies, which induced the birth of homogeneous liquid mixing of NR and single-layer Ti_3_C_2_T_x_ MXene. The mixture rapidly co-coagulated during emulsion flocculation, thereby constructing the filler network. The final vulcanisation process promoted the formation of sulphide cross-links between the rubber chains, further strengthening the network in MNx. After filling the NR with 1 wt% Ti_3_C_2_T_x_ MXene, the breakthrough time (BTT) of the composite to toluene, dichloromethane, and concentrated sulfuric acid increased by 140%, 178.6%, and 92.5%, respectively. Moreover, a striking improvement in the mechanical properties was also realized. The substantial optimization of the toxic chemical barrier and mechanical behaviours of NR is mainly due to the interface constructed between the Ti_3_C_2_T_x_ filler and NR matrix, which induced the formation of a robust 3D network in the composite.

## 2. Materials and Methods

### 2.1. Materials

Ti_3_AlC_2_ was provided by Jilin Yiyi Technology Co. (Jilin, China). A total of 38% hydrochloric acid (HCl) and lithium fluoride (LiF) were obtained from Beijing J&K Scientific Technology Co., Ltd. (Beijing, China) and Sinopharm Chemical Reagent Co. (Shanghai, China), respectively. NR emulsion (60 wt%) (solid content: 60 wt %) was purchased from Guangzhou Songbai Chemical Co., Ltd., (Changzhou, China). Calcium chloride (CaCl_2_, AR) was purchased from the Beijing Chemical Factory (Beijing, China). Rubber additives were commercial products. Other reagents were chemically pure and used as received.

### 2.2. Preparation of Ti_3_C_2_T_x_ MXene Nanosheets

**Figure 1:**
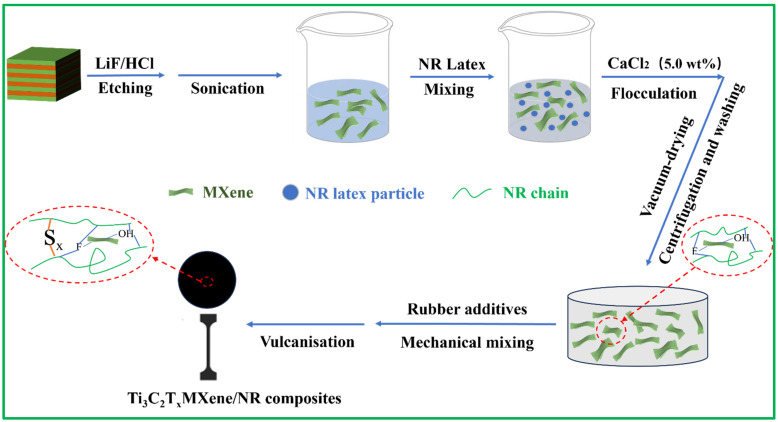
Schematic of the preparation of Ti_3_C_2_T_x_ MXene nanosheets and MNx composites.

As shown in Figure 1, Ti_3_C_2_T_x_ MXene sheets were synthesized by etching the Ti_3_AlC_2_ MAX phase with HCl/LiF solvents followed by ultrasonic delamination [28]. Then, the resulting suspension was rigorously washed with ultra-pure water. After freeze-drying for 6 h under a vacuum, the Ti_3_C_2_T_x_ MXene powder was dispersed in the aqueous phase via sonication to prepare a suspension of Ti_3_C_2_T_x_ MXene at a concentration of 20 mg/mL. Details are shown in the supporting information.

### 2.3. Preparation of MNx Composites

As described in Figure 1, the NR emulsion (60 wt%) was mixed with different contents of the above Ti_3_C_2_T_x_ MXene suspension (20 mg/mL) via continuous magnetic stirring for 4 h to obtain a uniform mixture. The detailed proportions of Ti_3_C_2_T_x_ and NR are shown in Table S1. Then, a CaCl_2_ solution (5 wt%) was added dropwise to the mixture for flocculation. Next, the completely flocculated mixture was precipitated with deionized water and filtered 3 times. Finally, the Ti_3_C_2_T_x_ MXene/NR masterbatch was obtained by freeze-drying the mixture under a vacuum for subsequent use.

Ti_3_C_2_T_x_ MXene/NR masterbatch was combined with additives on an open refining machine in accordance with the specific proportions, as depicted in Table S2. Vulcanised MNx with a thickness of 1 mm was achieved by plate vulcanisation at 143 °C for 10 min. The final nanocomposites with 0, 0.5, 1, and 3 wt% of Ti_3_C_2_T_x_ were named MN0, MN 0.5, MN1, and MN3, respectively.

### 2.4. Characterization

Morphological information was collected from a scanning electron microscope (SEM, ZEISS Gemini SEM 300, Jena, Germany). The crystal information was determined using an X-ray diffractometer (Bruker D8 Advance TXS) with Cu-Kα radiation. X-ray photoelectron spectroscopy (XPS) spectra were recorded using a Thermo Scientific K-Alpha (Thermo Electron Corporation, Waltham, MA, USA) X-ray photoelectron spectrometer with monochromatic Al-Kα (1486.6 eV) radiation. A rubber processing analyser (RPA) (RPA 2000, Alpha Technologies Corporation, Howell, MI, USA) was employed to monitor the relationship between storage modulus and the strain of uncured rubber compounds. TEM measurement was conducted on a JEOL JEM-2100 Plus transmission electron microscope (Japan Electronics Corporation, Tokyo, Japan) operated at 300 kV. And samples were embedded and sectioned using a Leica EM UC7 frozen ultrathin sectioning machine before TEM characterization. Atomic Force microscopy (AFM) test was carried out on a Bruker Multimode 8 with a trapping mode. Tensile testing was operated on a UCAN UT-2060 according to ASTM D 412. Dynamic mechanical analysis (DMA, Frequency: 1 Hz.) was performed on a TAQ 800 (TA Corporation, Santa Fe Springs, CA, USA) instrument in a tensile mode. Fourier transform infrared spectroscopy (FTIR) spectra were recorded using Nicolet iS20 Fourier transform infrared spectrometer (FT-IR, Thermo Scientific, Waltham, MA, USA). The cross-linking densities (CDs) of the vulcanisates were determined via nuclear magnetic resonance (NMR) spectroscopy (VTMR20-010V-1, Suzhou Niumag Corporation, Suzhou, China) at a frequency of 15 MHz, magnetic induction intensity of 0.5 ± 0.05 T and temperature of 90 °C. The bound rubber content in the MNx compounds was measured using a method reported by Leblanc [29]. Toxic chemical permeability tests were conducted at 25 °C using an apparatus for measuring chemical permeability in accordance with ISO 1817:2022. The tensile test and tear test were performed following ISO 37:2005 and ISO 34-1:2004 standards, respectively.

## 3. Results and Discussion

### 3.1. Microstructure of Ti_3_C_2_T_x_ MXene Nanosheets

**Figure 2:**
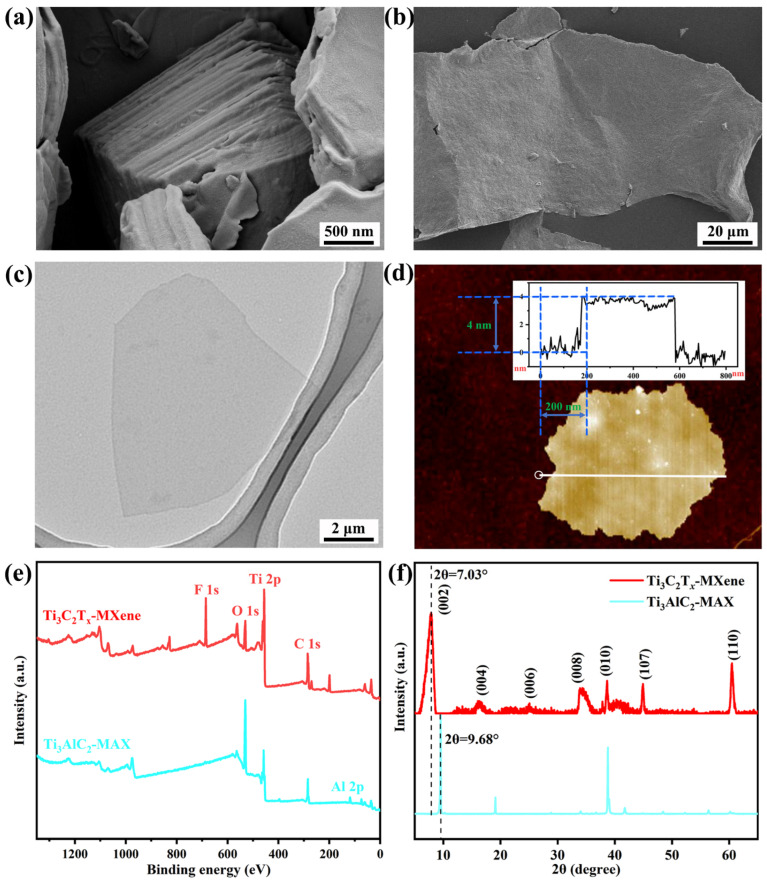
(**a**) SEM images of Ti_3_AlC_2_ MAX and (**b**) monolayer Ti_3_C_2_T_x_ MXene. (**c**) TEM and (**d**) AFM images of monolayer Ti_3_C_2_T_x_ MXene. (**e**) XPS survey spectra and (**f**) XRD patterns of Ti_3_AlC_2_ MAX and Ti_3_C_2_T_x_ MXene.

The SEM image in Figure 2a implies the tightly packed structure of the Ti_3_AlC_2_ MAX. After being etched by the LiF/HCl system, Ti_3_AlC_2_ MAX transformed into a loose accordion-like structure (Figure S1). The final ultrasonication step peeled off the loose Ti_3_AlC_2_ MAX into ultra-thin Ti_3_C_2_T_x_ Mxene sheets [Figure 2b,c]. The AFM image in Figure 2d suggests that the Ti_3_C_2_T_x_ MXene is 4 nm thick. The XPS survey spectra of Ti_3_AlC_2_ MAX and Ti_3_C_2_T_x_ MXene in Figure 2e show the appearance of C 1s, O 1s, and Ti 2p peaks in both. Of note, the enhancement of the Ti 2p signal, disappearance of the Al 2p peak, and occurrence of the F 1s peak in Ti_3_C_2_T_x_ MXene preliminarily confirmed the successful fabrication of Ti_3_C_2_T_x_ MXene. The XRD patterns in Figure 2f further confirmed the successful transformation of Ti_3_AlC_2_ MAX (JCPDS No. 52-0875) into Ti_3_C_2_T_x_ MXene [30,31]. Obviously, after the removal of the Al layer, peaks of the (002) pattern shifted from 9.68° to 7.03°. The disappearance of the strong and distinguished peak at 38.8° indicates that the accordion-like structure was separated into single-layer sheets.

### 3.2. Filler Dispersion of MNx Composites

**Figure 3:**
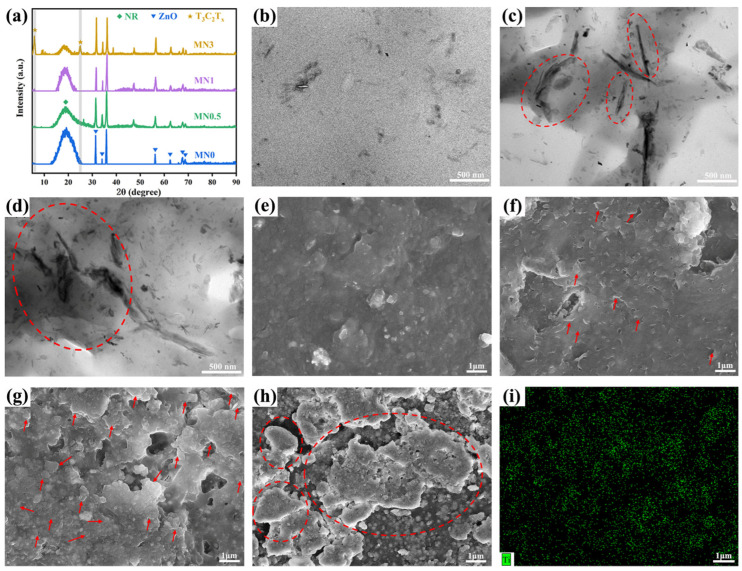
(**a**) XRD patterns of the MNx nanocomposites (x = 0, 0.5, 1, 3). TEM images of (**b**) MN0, (**c**) MN1, and (**d**) MN3. SEM images of MNx, (**e**) x = 0, (**f**) 0.5, (**g**) 1, (**h**) 3, and (**i**) SEM EDS elemental mapping of Ti elements for MN1.

Glue liquid mixing and subsequent flocculation solidification technologies were employed to manufacture the Ti_3_C_2_T_x_ MXene/NR (MNx, x = 0~3) composite. XRD was employed to examine the chemical compositions of MNx composites as well as confirm the uniformity of the Ti_3_C_2_T_x_ MXene in the NR matrix [Figure 3a]. Broden diffraction peaks at about 20° of all samples indicate the amorphous structure of the NR. The diffraction peaks between 30° and 70° were attributed to vulcanising auxiliary ZnO [32]. In the XRD patterns of MN0.5 and MN1, the lack of obvious characteristic peaks related to Ti_3_C_2_T_x_ MXene confirmed the uniform dispersion of Ti_3_C_2_T_x_ MXene throughout the entire NR matrix [33,34]. In contrast, the appearance of evident peaks of (002) and (004) patterns in the XRD spectrum of MN3 implied the aggregation of Ti_3_C_2_T_x_ Mxene sheets.

The uniform dispersion of the filler and interfacial interaction between filler and rubber are the key factors in determining the final properties of rubber composites [35]. Figure 3e–h show the SEM images of the MNx composites with different contents of Ti_3_C_2_T_x_ MXene. Actually, the surface of NR was flat and smooth [Figure 3e]. After being filled with Ti_3_C_2_T_x_ MXene, an obvious contour of the layered material was observed on the surface of MN0.5 to MN1 [Figure 3f,g]. Furthermore, Ti_3_C_2_T_x_ MXene sheet fillers were uniformly dispersed in the NR matrix with fewer defects on their surfaces. The SEM EDS elemental mapping of Ti elements in Figure 3i further demonstrates the uniform distribution of Ti_3_C_2_T_x_ MXene sheets. As x further increased to 3, large Ti_3_C_2_T_x_ MXene lamellar aggregates appeared and the number of defects increased significantly on the surface of the composites [Figure 3h], indicating the enrichment of Ti_3_C_2_T_x_ within the MNx composite.

The local dispersion of the Ti_3_C_2_T_x_ MXene nanosheets in the NR composites was analysed using TEM. The TEM images of MN0 and MN1 [Figure 3b,c] further illustrate the uniform distribution of Ti_3_C_2_T_x_ Mxene sheets in the NR, which may be due to the hydrogen bonds between the functional groups on the surface of Ti_3_C_2_T_x_ MXene and the molecular chains of NR. However, coarse dark lines in the NR matrix of MN3 [Figure 3d] imply the accumulation of Ti_3_C_2_T_x_ MXene nanosheets, attributed to the significant difference between the surface energy of Ti_3_C_2_T_x_ MXene and NR [36].

### 3.3. The Filler Network and Interfacial Interaction in MNx Composites

**Figure 4:**
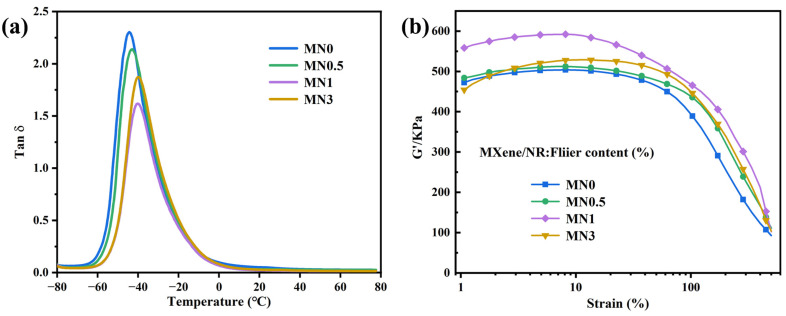
(**a**) Tan δ versus temperature curves and (**b**) plots of strain-dependent G’ for MN0, MN0.5, MN1.0, and MN3.

The interfacial interaction between the Ti_3_C_2_T_x_ MXene sheets and NR was studied using DMA. The loss factor (tan δ) at the glass transition temperature (*T*_g_) was inversely proportional to the volume of the constrained polymer chains [37]. Figure 4a shows tan δ as a function of temperature curves for the MNx composites obtained from DMA. As x increased, the tan δ at *T*_g_ first decreased and then increased and the inflection point appeared when *x* = 1. The preliminary downshift implied that more rubber chains were constrained by Ti_3_C_2_T_x_. This is reasonably explained by the 3D network constructed by the filler of Ti_3_C_2_T_x_ MXene restricting the mobility of NR molecules because of the hydrogen bond between Ti_3_C_2_T_x_ and the rubber chains. Such limitations enhanced the mechanical properties of MNx composite and its resistance against toxic chemicals [38,39]. When x further increased, Ti_3_C_2_T_x_ MXene aggregated and the limitation effect decreased, eventually resulting in an increase in tan δ at *T*_g_. The structure of the filler in the composites was analysed via dynamic rheological measurement. Figure 4b shows the strain-dependent storage modulus (G’) plots for uncured MNx (x = 0, 0.5, 1, and 3). As x increased to 1, the initial G’ value of Ti_3_C_2_T_x_ MXene/NR first slowly and then rapidly increased, which was attributed to the hydrodynamic effects resulting from the stiff fillers in the rubber matrix [40,41]. The failure to obtain a higher initial G’ when x = 3 may have resulted from the accumulation of Ti_3_C_2_T_x_ MXene in the composites. Such aggregation destructs the partial network of the Ti_3_C_2_T_x_ MXene nanosheets and thus weakens the interaction between Ti_3_C_2_T_x_ MXene and NR. The inverse relationship between the G’ value and strain is defined as the ‘‘Payne effect”, which is largely attributed to the damage of the filler network and the liberation of the constrained rubber chains in the filler network during oscillatory shear. The “Payne effect” of MN1 is the most obvious among all components, suggesting the formation of a highly interconnected 3D network, which would enhance the overall performance of MNx, particularly in terms of barrier properties [21].

### 3.4. Mechanism of Interfacial Interaction between Ti_3_C_2_T_x_ MXene Nanosheets and NR Molecules

**Figure 5:**
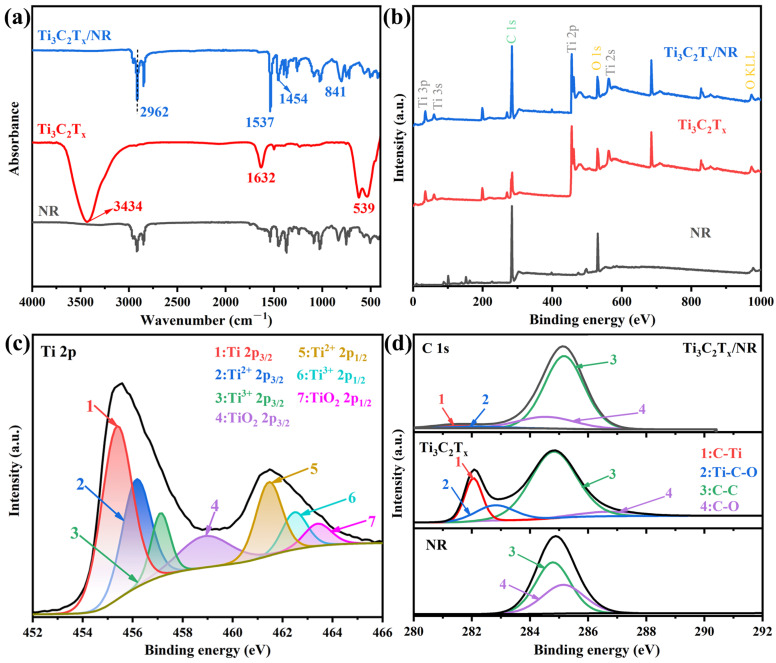
(**a**) FITR spectra and (**b**) XPS survey scans of NR, Ti_3_C_2_T_x_ MXene, and Ti_3_C_2_T_x_/NR. (**c**) XPS spectrum of Ti 2p in Ti_3_C_2_T_x_/NR. (**d**) C 1s spectra of NR, Ti_3_C_2_T_x_, and Ti_3_C_2_T_x_/NR composites.

The intense interaction between the NR matrix and Ti_3_C_2_T_x_ MXene filler is the key to establishing a continuous and stable network in the composite. Figure 5a exhibits the FT-IR spectra of NR, Ti_3_C_2_T_x_ Mxene, and Ti_3_C_2_T_x_ Mxene/NR. The typical signals of Ti_3_C_2_T_x_ Mxene are situated at 3434 cm^−1^ (–OH), 1632 cm^−1^ (=O), and 539 cm^−1^ (–OH) [42,43,44]. Three peaks related to the NR material located at 1454 cm^−1^, 841 cm^−1^, and 2949 cm^−1^, corresponding to the rotating vibration peak of –CH_3_, the out-of-plane oscillation peak of =CH–, and the asymmetric stretching vibration peak of –CH_3_, respectively. The concurrence of peaks for Ti_3_C_2_T_x_ MXene and NR in the FT-IR spectrum of Ti_3_C_2_T_x_/NR indicates that these two components coexist stably in the obtained Ti_3_C_2_T_x_/NR composites. Notably, the asymmetric stretching peak of –CH_3_ in the Ti_3_C_2_T_x_/NR composites shifts from 2949 cm^−1^ to 2962 cm^−1^ compared to pure NR, implying the existence of an interaction between the methyl group on the NR molecular chain and the surface functional group of Ti_3_C_2_T_x_. Actually, permanent dipole attraction and the presence of oxygen bonds can promote the surface hydration of rubber particles [45]. The above results confirm the formation of hydrogen bonds between the hydrated surface of NR and the polar groups (–OH and –F) of Ti_3_C_2_T_x_. These hydrogen bonds enhance the interfacial interaction between Ti_3_C_2_T_x_ MXene sheets and the NR matrix, resulting in uniform dispersion of Ti_3_C_2_T_x_ MXene nanosheets in the NR matrix and holds promise for improving the mechanical and toxic chemical barrier properties of the Ti_3_C_2_T_x_/NR composite material.

XPS analysis was conducted to further verify the interface between Ti_3_C_2_T_x_ MXene and NR. As shown in Figure 5b, both of the characteristic signals of Ti_3_C_2_T_x_ and NR appear in the XPS survey spectrum of Ti_3_C_2_T_x_/NR. After hybridization, the atomic ratios of Ti/C and Ti/O decreases significantly (Ti_3_C_2_T_x_/NR: Ti/C = 0.09 and Ti/O = 1.21; Ti_3_C_2_T_x_: Ti/C = 0.96 and Ti/O = 1.45). In the Ti 2p spectrum of Ti_3_C_2_T_x_/NR [Figure 5c], obvious C-Ti-T_x_ 2p_3/2_ and C-Ti-T_x_ 2p_1/2_ peaks indicate that Ti_3_C_2_T_x_ interacts with NR in the composite. Additionally, the C 1s spectrum of Ti_3_C_2_T_x_/NR [Figure 5d] shows the characteristic peaks of C-Ti-T_x_ [46] from Ti_3_C_2_T_x_ MXene and intense C–C and C–O peaks from NR with polar groups and electronegative elements [45,47,48]. These polar groups and electronegative elements guarantee the steady distribution of rubber latex in the aqueous solution [45]. The action of permanent dipole attraction and oxygen bonds are able to promote the surface of the rubber particles to be hydrated. Hence, it is anticipated that the strong interaction between the Ti_3_C_2_T_x_ MXene nanosheets and NR originates from the hydrogen bonds generated between the –OH and =O groups on Ti_3_C_2_T_x_ MXene and the C–O groups on NR. For the composite, the C-Ti-T_x_ peak shifts to a lower binding energy compared with that of Ti_3_C_2_T_x_ [49]_,_ confirming the formation of hydrogen bonds in the composites. Overall, the interface constructed between the Ti_3_C_2_T_x_ MXene nanosheet filler and NR matrix forms a high-quality network in the nanocomposites, which optimizes the mechanical and barrier behaviours of MN_x_ hybrids.

### 3.5. Bound Rubber Contents and Crosslinking Densities of MNx Composites

Table 1 displays the contents of bound rubber and CDs of MNx. The rubber combined with the filler prior to crosslinking is defined as bound rubber and mainly results from interfacial interactions between the fillers and rubber molecules. Compared with pure NR, the combination of NR with Ti_3_C_2_T_x_ enhances the content of bound rubber significantly, confirming the existence of strong interfacial interactions between Ti_3_C_2_T_x_ and adjacent NR molecules. This observation is consistent with the rheological tests depicted in Figure 4b. The enhanced CD after the combination of NR with Ti_3_C_2_T_x_ benefits from two factors: (1) the optimized crosslinking network induced by intense interactions, such as hydrogen bonds between the Ti_3_C_2_T_x_ and NR molecules; (2) the general sulphur crosslinking of the network. The enhanced CD of MNx composites benefits from the reduction in the distance among NR molecules, thereby optimizing the barrier behaviour [50].

### 3.6. Barrier Properties of MNx Composites

**Figure 6:**
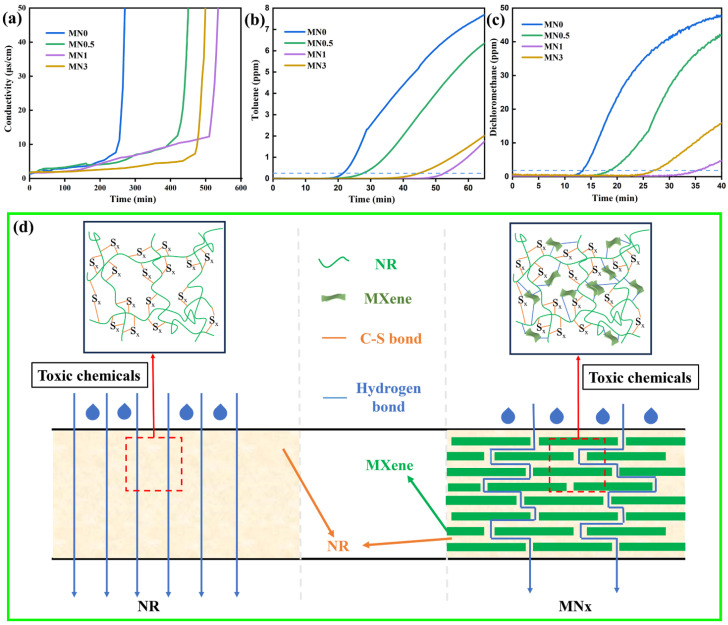
Permeability of MNx composites (x = 0, 0.5, 1, and 3) for (**a**) concentrated H_2_SO_4_, (**b**) toluene, and (**c**) dichloromethane. (**d**) Schematic illustration for the diffusion of the toxic chemicals in NR and MNx.

It remains a challenge to achieve excellent chemical protection properties of polymer composites with low content of Ti_3_C_2_T_x_ MXene. Figure 6a–c show the BTT of various toxic chemicals to MNx. The BTT of MNx to toluene, dichloromethane, and concentrated sulfuric acid gradually increased with an increase in x until x = 1, whereas the BTT was enhanced by 140%, 178.6%, and 92.5%. The delay in the penetration process of the MNx composites is attributed to two factors, as shown in Figure 6d. First, the free volume of NR molecular chains shrinks significantly because of the intricate 3D network of Ti_3_C_2_T_x_ MXene sheet filler and strong interfacial interaction between the Ti_3_C_2_T_x_ and NR molecules. Second, the 2D structure of Ti_3_C_2_T_x_ MXene nanosheets extends the diffusion pathway of harmful chemicals in the NR matrix. Composites with negligible amounts of Ti_3_C_2_T_x_ MXene have a weak blocking impact on rubber chain slip and interface interactions, whereas excessive Ti_3_C_2_T_x_ MXene accumulates in the composites, decreasing its blocking impact and increasing flaws in the composite. To sum up, the eye-catching chemical protection property was achieved in MNx composites with merely 1 wt% Ti_3_C_2_T_x_ MXene. As shown in Table S4, the chemical protection properties of this work (the BTT of MN1 to toluene and dichloromethane) significantly improved, compared with those of the published materials, especially for the innovative precise characterization of the corrosion resistance of concentrated sulfuric acid [51,52].

### 3.7. Mechanical properties of MNx composites

**Figure 7:**
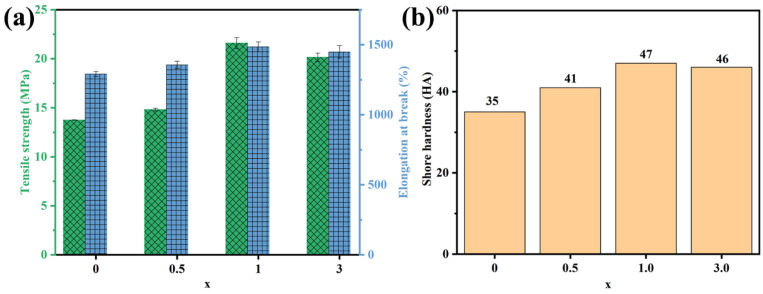
Mechanical properties of MNx system (x = 0~3): (**a**) tensile strength, elongation at break, and (**b**) shore hardness as a function of x curves.

Excellent stretchability, abrasion resistance, and flexural flexibility guarantee the proper functioning of the CPC in practical applications. The evolutions of the tensile strength and elongation at the break of the MNx system are shown in Figure 7a, and other mechanical properties are exhibited in Table S3. After the combination of Ti_3_C_2_T_x_ with NR, the tensile strength, elongation at break, and modulus at 100% and 300% strain of the MNx (x = 0.5~3) significantly improved in comparison with bare NR, especially for MN1 (increased by 7.847 MPa, 194% and 12 HA). It is the strong interfacial interaction that efficiently accelerated the movement of stress from the NR to the 2D Ti_3_C_2_T_x_ MXene nanosheets. However, the incorporation of excessive Ti_3_C_2_T_x_ MXene weakened the mechanical properties of MNx, mainly due to the increase in defects as well as a decrease in hydrogen bonds resulting from the aggregation of Ti_3_C_2_T_x_ in the NR matrix.

## 4. Conclusions

In summary, an MNx composite was successfully synthesized via a combination of latex mixing, emulsion flocculation, and flat-plate vulcanisation technologies. Due to the strong interfacial interactions between the Ti_3_C_2_T_x_ MXene filler and NR matrix, a high-quality 3D network forms in the nanocomposites and it highly optimizes the toxic chemical barrier and mechanical behaviours of NR. The nanocomposite with 1 wt% Ti_3_C_2_T_x_ MXene nanosheets achieves a substantial enhancement of BTT to toluene, dichloromethane, and concentrated sulfuric acid when compared with those of pure NR (increased by 140%, 178.6%, and 92.5%, respectively). Furthermore, the tensile strength, elongation at break, and shore hardness of MN1 were increased by 7.847 MPa, 194%, and 12 HA, respectively, compared with MN0. This study not only implies Ti_3_C_2_T_x_ MXene/NR as a potential CPC material but also provides a universal strategy to design multifunctional MXene/rubber composites for strain sensors and other applications.

## Figures and Tables

**Table 1:** Bound rubber content and CD of NR composites.

Samples	MN0	MN0.5	MN1	MN3
Bound rubber content (%)	-	0.58 ± 0.03	1.53 ± 0.05	4.02 ± 0.16
CD (×10^−4^ mol/mL)	10.27 ± 0.03	11.36 ± 0.05	13.90 ± 0.04	11.27 ± 0.06

## Data Availability

All information is available within the article.

## Supplementary Materials

The following supporting information can be downloaded at: https://www.mdpi.com/article/10.3390/polym15214260/s1, Figure S1: SEM images of Ti_3_AlC_2_ MAX with loose accordion-like structure; Table S1: Composition ratio design of Ti_3_C_2_T_x_ MXene/NR; Table S2: Test Prescription; Table S3: Mechanical properties of NR composites; Table S4: The chemical protection properties of rubber and rubber composites.

## References

[1] Wang R., Xu K., Xu Y., Wu Y. (2020). Study on prediction model of hazardous chemical accidents. J. Loss Prev. Process Ind..

[2] Wang B., Li D., Wu C. (2020). Characteristics of hazardous chemical accidents during hot season in China from 1989 to 2019: A statistical investigation. Saf. Sci..

[3] Mercey G., Verdelet T., Renou J., Kliachyna M., Baati R., Nachon F., Jean L., Renard P.-Y. (2012). Reactivators of Acetylcholinesterase Inhibited by Organophosphorus Nerve Agents. Accounts Chem. Res..

[4] Rosman Y., Eisenkraft A., Milk N., Shiyovich A., Ophir N., Shrot S., Kreiss Y., Kassirer M. (2014). Lessons Learned From the Syrian Sarin Attack: Evaluation of a Clinical Syndrome Through Social Media. Ann. Intern. Med..

[5] Kim Y., Choi M., Heo J., Jung S., Ka D., Jung H., Lee S., Jin Y., Hong J. (2021). Effect of surface charge density of graphene oxide on chemical warfare agent simulants blocking. Appl. Surf. Sci..

[6] Wisniewski A., Pirszel J. (2020). Protection of armoured vehicles against chemical, biological and radiological contamination. Def. Technol..

[7] Mao N., Lawrence C.A. (2014). 3-High performance textiles for protective clothing. High Performance Textiles and Their Applications.

[8] Bensel C.K. (1993). The effects of various thicknesses of chemical protective gloves on manual dexterity. Ergonomics.

[9] Kincl L.D., Bhattacharya A., Succop P.A., Clark C.S. (2002). Postural Sway Measurements: A Potential Safety Monitoring Technique for Workers Wearing Personal Protective Equipment. Appl. Occup. Environ. Hyg..

[10] Rothe M.J. (2002). Hand eczema. 2nd edition. J. Am. Acad. Dermatol..

[11] Bhuiyan M.A.R., Wang L., Shaid A., A Shanks R., Ding J. (2018). Advances and applications of chemical protective clothing system. J. Ind. Text..

[12] Zhang N., Pang Y., Li Z., Yang C., Zong L., Yang H., Wu H., Duan Y., Zhang J. (2023). Rubber-like and biodegradable poly (vinyl alcohol) composites with triple networks for high-efficiency solvent barrier. Compos. Sci. Technol..

[13] Jose S., Thomas S., Jibin K., Sisanth K., Kadam V., Shakyawar D. (2022). Surface modification of wool fabric using sodium lignosulfonate and subsequent improvement in the interfacial adhesion of natural rubber latex in the wool/rubber composites. Ind. Crop. Prod..

[14] Kim K., Jung H., Cho K.M. (2023). ZIF-8/Graphene Oxide Hybrid Membranes as Breathable and Protective Barriers against Chemical Warfare Agents. ACS Appl. Mater. Interfaces.

[15] Ren X., Barrera C.S., Tardiff J.L., Gil A., Cornish K. (2020). Liquid guayule natural rubber, a renewable and crosslinkable processing aid in natural and synthetic rubber compounds. J. Clean. Prod..

[16] Wang Q., Sun T., Qiao Y., Liu H., Zhang C., Zhang X., Sun Y. (2023). Effects of microstructures of liquid polyisoprene on the properties of styrene–butadiene rubber/butadiene rubber compounds. Polym. Int..

[17] Jayathilaka L.P.I., Ariyadasa T.U., Egodage S.M. (2020). Development of biodegradable natural rubber latex composites by employing corn derivative bio-fillers. J. Appl. Polym. Sci..

[18] George N., Varghese G.A., Joseph R. (2019). Improved mechanical and barrier properties of Natural rubber-Multiwalled carbon nanotube composites with segregated network structure. Mater. Today Proc..

[19] Nouraei M., Liaghat G., Ahmadi H., Bahramian A.R., Taherzadeh-Fard A., Vahid S. (2022). High strain-rate and quasi-static mechanical characteristics of the natural rubber-based elastomer nanocomposite reinforced with alumina nanoparticles. J. Reinf. Plast. Compos..

[20] Wongwat S., Yoksan R., Hedenqvist M.S. (2022). Bio-based thermoplastic natural rubber based on poly(lactic acid)/thermoplastic starch/calcium carbonate nanocomposites. Int. J. Biol. Macromol..

[21] Li Q., Zhong B., Zhang W., Jia Z., Jia D., Qin S., Wang J., Razal J.M., Wang X. (2019). Ti_3_C_2_ MXene as a new nanofiller for robust and conductive elastomer composites. Nanoscale.

[22] Naguib M. (2016). Cation Intercalation and High Volumetric Capacitance of Two-Dimensional Titanium Carbide. Science.

[23] Peng W., Han J., Lu Y.-R., Luo M., Chan T.-S., Peng M., Tan Y. (2022). A General Strategy for Engineering Single-Metal Sites on 3D Porous N, P Co-Doped Ti_3_C_2_T_X_ MXene. ACS Nano.

[24] Xue R., Wang C.-X., Zhao Z.-G., Chen Y.-H., Yang J., Feng C.-P. (2023). Flexible Silica/MXene/Natural rubber film strain sensors with island chain structure for Healthcare monitoring. J. Colloid Interface Sci..

[25] Xu W., Su J., Lin J., Huang J., Weng M., Min Y. (2023). Enhancing the light-thermal absorption and conversion capacity of diatom-based biomass/polyethylene glycol composites phase change material by introducing MXene. J. Energy Storage.

[26] Han R., Xie Y., Ma X. (2018). Crosslinked P84 copolyimide/MXene mixed matrix membrane with excellent solvent resistance and permselectivity. Chin. J. Chem. Eng..

[27] Aakyiir M., Oh J.-A., Araby S., Zheng Q., Naeem M., Ma J., Adu P., Zhang L., Mai Y.-W. (2021). Combining hydrophilic MXene nanosheets and hydrophobic carbon nanotubes for mechanically resilient and electrically conductive elastomer nanocomposites. Compos. Sci. Technol..

[28] Ghidiu M., Lukatskaya M.R., Zhao M.-Q., Gogotsi Y., Barsoum M.W. (2014). Conductive two-dimensional titanium carbide ‘clay’ with high volumetric capacitance. Nature.

[29] Baumann W., Ismeier M., Baumann W., Ismeier M. (1998). Grundlagen zu Kautschuk. Kautschuk und Gummi: Daten und Fakten zum Umweltschutz Band ½.

[30] Ng V.M.H., Huang H., Zhou K., Lee P.S., Que W., Xu J.Z., Kong L.B. (2016). Recent progress in layered transition metal carbides and/or nitrides (MXenes) and their composites: Synthesis and applications. J. Mater. Chem. A.

[31] Alhabeb M., Maleski K., Anasori B., Lelyukh P., Clark L., Sin S., Gogotsi Y. (2017). Guidelines for Synthesis and Processing of Two-Dimensional Titanium Carbide (Ti3C2T00000000 MXene). Chem. Mater..

[32] Xu Z., Zheng L., Wen S., Liu L. (2019). Graphene oxide-supported zinc oxide nanoparticles for chloroprene rubber with improved crosslinking network and mechanical properties. Compos. Part A Appl. Sci. Manuf..

[33] Yin B., Zhang X., Wang J., Wen Y., Jia H., Ji Q., Ding L. (2017). Ionic liquid functionalized graphene oxide for enhancement of styrene-butadiene rubber nanocomposites. Polym. Adv. Technol..

[34] Yang Z., Liu H., Wu S., Tang Z., Guo B., Zhang L. (2018). A green method for preparing conductive elastomer composites with interconnected graphene network via Pickering emulsion templating. Chem. Eng. J..

[35] Punetha V.D., Rana S., Yoo H.J., Chaurasia A., McLeskey J.T., Ramasamy M.S., Sahoo N.G., Cho J.W. (2017). Functionalization of carbon nanomaterials for advanced polymer nanocomposites: A comparison study between CNT and graphene. Prog. Polym. Sci..

[36] Tang Z., Zhang L., Feng W., Guo B., Liu F., Jia D. (2014). Rational Design of Graphene Surface Chemistry for High-Performance Rubber/Graphene Composites. Macromolecules.

[37] Rao J.M. (2007). Pochan, Mechanics of Polymer−Clay Nanocomposites. Macromolecules.

[38] Angellier H., Molina-Boisseau S., Dufresne A. (2005). Mechanical Properties of Waxy Maize Starch Nanocrystal Reinforced Natural Rubber. Macromolecules.

[39] Das A., Kasaliwal G.R., Jurk R., Boldt R., Fischer D., Stöckelhuber K.W., Heinrich G. (2012). Rubber composites based on graphene nanoplatelets, expanded graphite, carbon nanotubes and their combination: A comparative study. Compos. Sci. Technol..

[40] Sorokin V.V., Ecker E., Stepanov G.V., Shamonin M., Monkman G.J., Kramarenko E.Y., Khokhlov A.R. (2014). Experimental study of the magnetic field enhanced Payne effect in magnetorheological elastomers. Soft Matter.

[41] Gan S., Wu Z.L., Xu H., Song Y., Zheng Q. (2016). Viscoelastic Behaviors of Carbon Black Gel Extracted from Highly Filled Natural Rubber Compounds: Insights into the Payne Effect. Macromolecules.

[42] Luo J.-Q., Zhao S., Zhang H.-B., Deng Z., Li L., Yu Z.-Z. (2019). Flexible, stretchable and electrically conductive MXene/natural rubber nanocomposite films for efficient electromagnetic interference shielding. Compos. Sci. Technol..

[43] Sun R., Zhang H., Liu J., Xie X., Yang R., Li Y., Hong S., Yu Z. (2017). Highly Conductive Transition Metal Carbide/Carbonitride(MXene)@polystyrene Nanocomposites Fabricated by Electrostatic Assembly for Highly Efficient Electromagnetic Interference Shielding. Adv. Funct. Mater..

[44] Boota M., Anasori B., Voigt C., Zhao M.-Q., Barsoum M.W., Gogotsi Y. (2015). Pseudocapacitive Electrodes Produced by Oxidant-Free Polymerization of Pyrrole between the Layers of 2D Titanium Carbide (MXene). Adv. Mater..

[45] Jacob J.-L., D’auzac J., Prevôt J.-C. (1993). The composition of natural latex fromHevea brasiliensis. Clin. Rev. Allergy.

[46] Halim J., Cook K.M., Naguib M., Eklund P., Gogotsi Y., Rosen J., Barsoum M.W. (2016). X-ray photoelectron spectroscopy of select multi-layered transition metal carbides (MXenes). Appl. Surf. Sci..

[47] Zhao S., Zhang H.-B., Luo J.-Q., Wang Q.-W., Xu B., Hong S., Yu Z.-Z. (2018). Highly Electrically Conductive Three-Dimensional Ti3C2Tx MXene/Reduced Graphene Oxide Hybrid Aerogels with Excellent Electromagnetic Interference Shielding Performances. ACS Nano.

[48] Yan N., Buonocore G., Lavorgna M., Kaciulis S., Balijepalli S.K., Zhan Y., Xia H., Ambrosio L. (2014). The role of reduced graphene oxide on chemical, mechanical and barrier properties of natural rubber composites. Compos. Sci. Technol..

[49] Bokobza L. (2019). Natural Rubber Nanocomposites: A Review. Nanomaterials.

[50] Zheng L., Jerrams S., Xu Z., Zhang L., Liu L., Wen S. (2019). Enhanced gas barrier properties of graphene oxide/rubber composites with strong interfaces constructed by graphene oxide and sulfur. Chem. Eng. J..

[51] Zheng L., Wang D., Xu Z., Zhang L., Liu L., Wen S. (2018). High barrier properties against sulfur mustard of graphene oxide/butyl rubber composites. Compos. Sci. Technol..

[52] De Kee D., Fong C.F.C.M., Pintauro P., Hinestroza J., Yuan G., Burczyk A. (2000). Effect of temperature and elongation on the liquid diffusion and permeation characteristics of natural rubber, nitrile rubber, and bromobutyl rubber. J. Appl. Polym. Sci..

