# Supplementary material for: Boosted Chemical Protective Properties Using Interface Constructed between Ti3C2Tx MXene and Natural Rubber
Source: Polymers (Basel). 2023 Oct 30;15(21):4260. doi: 10.3390/polym15214260 (PMC10648372; doi:10.3390/polym15214260)
Supplement: Supplementary file 1 [file polymers-15-04260-s001.zip › polymers-2656044-supplementary.pdf]

# Supplementary Information

**Preparation of $Ti_3C_2T_x$ MXene nanosheets**

MXene ($Ti_3C_2T_x$) nanosheets were obtained by selectively etching the $Ti_3AlC_2$ with LiF/HCl solution as described in previous publications. Typically, 2.0 g LiF and 40 mL 9 M HCl solution were mixed in a Teflon beaker with continually stirring to ensure the dissolution of LiF. Then, 2.0 g $Ti_3AlC_2$ powder was slowly added into the above mixture and stirred gently at 35 °C for 24 h to the etching of Al. The prepared suspension was repeatedly washed with deionized water through centrifugation at 3500 rpm until the pH value of supernatant was above 6. After that, the MXene sediment was dispersed in deionized water through ice-bath ultrasonication for 60 min to exfoliate the MXene flakes. Finally, a dark green homogeneous MXene nanosheets dispersion liquid was acquired by centrifugation at 3500 rpm for 1 h. The monolayer $Ti_3C_2T_x$ MXene is obtained after vacuum freeze-drying.

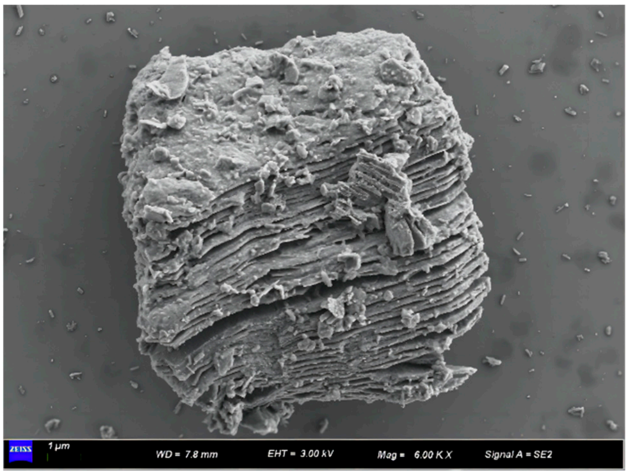


**Figure S1**. SEM images of $Ti_3AlC_2$ MAX with loose accordion-like structure.

**Table S1.** Composition ratio design of $Ti_3C_2T_x$ MXene/NR

| Group | Pure NR（g） | $Ti_3C_2T_x$ MXene suspension dosage（ml） | Content of $Ti_3C_2T_x$ MXene（wt%） |
|---|---|---|---|
| 1 | 166.67 | 0 | 0 |
| 2 | 165.83 | 25 | 0.5 |
| 3 | 165.00 | 50 | 1.0 |
| 4 | 163.00 | 150 | 3.0 |

**Table S2.** Test Prescription

| MNx (g) | ZnO（g） | SA（g） | DM（g） | D（g） | CZ（g） | M（g） | S（g） |
|---|---|---|---|---|---|---|---|
| 100 | 5.0 | 2.0 | 0.8 | 0.2 | 0.3 | 0.5 | 1.5 |

SA: stearic acid,

CZ: N-Cyclohexyl-2- benzothiazolesulfenamide，

DM: N,N- Dimethylaminoethyl Methacrylate,

D: D-Tartaric acid,

M: 2-Mercaptobenzothiazole.

We have considered the further controls and the specific improvements, which can regard the methodology. We believe that the dispersion of rubber additives could be intruded in latex mixing process rather than additional mechanical mixing process to shorten the synthetic period of $Ti_3C_2T_x$ MXene/NR composites.

Optimization plan:

Control factor 1: Uniform dispersion of rubber additives.

Optimization mode considered: rubber additives should be added in NR latex after emulsification rather than direct addition.

Control factor 2: The adding order of various rubber additives.

Optimization method considered: The sulfur should be added last to avoid early occurrence of vulcanization reaction during latex mixing.

**Table S3.** Mechanical properties of NR composites

| Content of $Ti_3C_2T_x$ MXene （wt%） | Tensile Strength (MPa) | Elongation at Break (100%) | Shore Hardness (HA) | Modulus at 100% strain (MPa) | Modulus at 300% strain (MPa) |
|---|---|---|---|---|---|
| 0 | 13.757±0.023 | 1291±18 | 35 | 0.7±0.1 | 1.5±0.1 |
| 0.5 | 14.816±0.138 | 1357±26 | 41 | 0.8±0.1 | 2.2±0.1 |
| 1.0 | 21.604±0.563 | 1485±35 | 47 | 0.9±0.1 | 2.0±0.1 |
| 3.0 | 20.155±0.428 | 1449±45 | 46 | 0.8±0.1 | 2.2±0.1 |

**Table S4.** The chemical protection properties of rubber and rubber composites.

| Sample | Toxic chemical | BTT (min) | Ref. |
|---|---|---|---|
| Butyl rubber (IIR) | Sulfur mustard | 830 | [1] |
| GO/IIR | Sulfur mustard | 928 | [1] |
| Nitrile rubber | Acetone | 13.2 | [2] |
| Nitrile rubber | Dichloromethane | 5.8 | [2] |
| Nitrile rubber | Toluene | 36.7 | [2] |
| Bromobutyl rubber | Dichloromethane | 16.5 | [2] |
| Bromobutyl rubber | Toluene | 23 | [2] |
| NR | Acetone | 34.7 | [2] |
| This work | Toluene | 52.8 | / |
| This work | Dichloromethane | 36.8 | / |
| This work | Concentrated sulfuric acid | 523 | / |

[1] L. Zheng, D. Wang, Z. Xu, L. Zhang, L. Liu, S. Wen, High barrier properties against sulfur mustard of graphene oxide/butyl rubber composites, Composites Science and Technology 170 (2019) 141-147. doi.org/Doi.

[2] D. De Kee, C.F.C. Man Fong, P. Pintauro, J. Hinestroza, G. Yuan, A. Burczyk, Effect of temperature and elongation on the liquid diffusion and permeation characteristics of natural rubber, nitrile rubber, and

bromobutyl rubber, Journal of Applied Polymer Science 78 (2000) 1250-1255.doi.org/Doi.
